# Transfer Learning-Based Multi-Scale Denoising Convolutional Neural Network for Prostate Cancer Detection

**DOI:** 10.3390/cancers14153687

**Published:** 2022-07-28

**Authors:** Kwok Tai Chui, Brij B. Gupta, Hao Ran Chi, Varsha Arya, Wadee Alhalabi, Miguel Torres Ruiz, Chien-Wen Shen

**Affiliations:** 1 Department of Electronic Engineering and Computer Science, School of Science and Technology, Hong Kong Metropolitan University, Hong Kong, China; 2 International Center for AI and Cyber Security Research and Innovations, Department of Computer Science and Information Engineering, Asia University, Taichung 41354, Taiwan; 3 Research and Innovation Department, Skyline University College, Sharjah P.O. Box 1797, United Arab Emirates; 4 Department of Computer Science, King Abdulaziz University, Jeddah 21589, Saudi Arabia; wsalhalabi@kau.edu.sa; 5 Lebanese American University, Beirut 1102, Lebanon; 6 Instituto de Telecomunicações, 3810-193 Aveiro, Portugal; ytchr@av.it.pt; 7 Insights2Techinfo, India; varsha.arya@insights2techinfo.com; 8 Instituto Politécnico Nacional, Centro de Investigacion en Computacion, UPALM-Zacatenco, Mexico City 07320, Mexico; mtorresru@ipn.mx; 9 Department of Business Administration, National Central University, Taoyuan City 320317, Taiwan; cwshen@ncu.edu.tw

**Keywords:** automatic diagnosis, convolutional neural network, deep learning, image denoising, prostate cancer, transfer learning

## Abstract

**Simple Summary:**

To enhance the automatic diagnosis of the prostate cancer using machine learning algorithm, we modify the design of convolutional neural network to support multi-scale denoising of cancer images. Transfer learning is employed to leverage the detection accuracy of the prostate cancer detection model by taking advantages from more unseen data from a source dataset. Compared to existing methodologies, our work improves the accuracy by more than 10%. Ablation studies have conducted to evaluate the contributions of the components of the proposed algorithm, with 2.80%, 3.30%, and 3.13% for image denoising, multi-scale scheme, and transfer learning, respectively. The results reveal the effectiveness of the algorithm and provide insights for five future research directions.

**Abstract:**

Background: Prostate cancer is the 4th most common type of cancer. To reduce the workload of medical personnel in the medical diagnosis of prostate cancer and increase the diagnostic accuracy in noisy images, a deep learning model is desired for prostate cancer detection. Methods: A multi-scale denoising convolutional neural network (MSDCNN) model was designed for prostate cancer detection (PCD) that is capable of noise suppression in images. The model was further optimized by transfer learning, which contributes domain knowledge from the same domain (prostate cancer data) but heterogeneous datasets. Particularly, Gaussian noise was introduced in the source datasets before knowledge transfer to the target dataset. Results: Four benchmark datasets were chosen as representative prostate cancer datasets. Ablation study and performance comparison between the proposed work and existing works were performed. Our model improved the accuracy by more than 10% compared with the existing works. Ablation studies also showed average improvements in accuracy using denoising, multi-scale scheme, and transfer learning, by 2.80%, 3.30%, and 3.13%, respectively. Conclusions: The performance evaluation and comparison of the proposed model confirm the importance and benefits of image noise suppression and transfer of knowledge from heterogeneous datasets of the same domain.

## 1. Introduction

The World Health Organization (WHO) has estimated that new cases of prostate cancer total more than 1.414 million annually [1]. It ranks 4th, 2nd, and 2nd based on the total number of new cases, crude rate, and age-standardized rate, respectively. Several measures were proposed to reduce the mortality rates of cancers such as the encouragement of cancer screening participation [2], healthy diet [3], and aligning with the sustainable development goals [4], which only contribute to a small extent. However, the world is facing two major challenges: (i) the worsening of population ageing, which will increase the prevalence of cancers and need for medical care [5,6]; and (ii) the long-standing issue of medical staff shortages, leading to heavier workloads and lowered productivity among medical staff due to multi-tasking [7,8].

The benefits of artificial intelligence in the healthcare industry were studied [9,10,11]. Automatic diagnosis of prostate cancer via machine learning models is expected to relieve the workload of medical staff and enhance detection accuracy. Positron emission tomography (PET), computed tomography (CT), and magnetic resonance imaging (MRI) scans are typical images to capture the information inside the body and thus help medical staff with cancer diagnosis. Noisy images can be observed in these images, where typical noises are Rayleigh, impulse, temporal, Gaussian, and Rician. Image noise suppression has become important before performing medical diagnosis. Particularly, the noise is heterogeneous (but similar) across datasets; however, borrowing knowledge from different benchmark datasets using transfer learning (TL) to the target dataset may help improve the prostate cancer detection (PCD) model. This provided the initiative in our work to propose a transfer learning-based multi-scale denoising convolutional neural network (TL-MSDCNN) model for PCD. Four benchmark prostate cancer datasets were selected for performance evaluation and analysis of the proposed model. They are NaF Prostate [12], TCGA-PRAD [13], Prostate-3T [14], and PROSTATE-DIAGNOSIS [15], which are publicly accessible from The Cancer Imaging Archive [16].

The structure of this paper is organized as follows. The first section comprises three subsections. Section 1.1 summarizes the methodologies and results of existing works. Section 1.2 presents the research limitations in the existing works. Section 1.3 highlights the research contributions of our work. The details of the four benchmark datasets and methodology of the proposed algorithm are presented in Section 2. This is followed by the performance evaluation of the proposed algorithm, its ablation study, and the comparison with existing works (those covered in Section 1.1). Section 4 details the ablation studies on the three components of the proposed algorithm: denoising, multi-scale scheme, and transfer learning. Lastly, in Section 5, a conclusion is drawn with some future research directions.

### 1.1. Methodologies and Results of Existing Works

To ensure that the performance evaluation and comparison in later sections are on the same page, the selected existing works [17,18,19,20,21,22,23,24] in this subsection utilized four benchmark datasets.

The discussion first starts with the NaF Prostate dataset. In [17], 172 probability features were extracted from PET/CT images to build a random forest classifier for PCD. The classifier achieved a sensitivity and specificity of 88% and 89%, respectively. Another work [18] employed TL to fine-tune the DenseNet-121 PCD model using pre-trained ImageNet. A sensitivity of 88% was observed.

In regard to the TCGA-PRAD dataset, a bag-of-features representation-based convolutional neural network (CNN) model was proposed for PCD [19]. It achieved an accuracy of 77%, which outperformed two existing works using GoogLeNet and Modified AlexNet by 0.13 and 4.73%, respectively. Another work [20] also employed CNN with the addition of a class activation map using global average pooling. In terms of performance, the model achieved sensitivity, specificity, and accuracy of 81.5%, 82%, and 81.75%, respectively.

Using the Prostate-3T dataset, the YOLO convolutional network was used with four segmentation techniques, namely morphological dilation, particle swarm optimization, ResCNN, and intrinsic manifold simple linear iterative clustering, to train the MRI scans slice by slice from the axial view [21]. As a preliminary study, small-scale subsets were used for performance evaluation. The sensitivity, specificity, and accuracy of the model were 88.4%, 93.4%, and 92.0%, respectively. As an extension from [21], pixels and superpixels were extracted from the MRI scans [22] and served as inputs for the CNN-based PCD. Probabilistic Atlas, intrinsic manifold simple linear iterative clustering, and particle swarm optimization were used to support the CNN algorithm. The model with former inputs obtained sensitivity, specificity, and accuracy of 76.3%, 96.3%, and 91.59%, respectively, whereas the latter inputs yielded 88.7%, 99.1%, and 98.7%, respectively.

With regard to the PROSTATE-DIAGNOSIS dataset, MRI super-resolution was considered in the MSG-GAN and CapsGAN model [23] for PCD. The accuracy of the model was 79% using only one-tenth of the available data in model training. Another work [24] proposed a super resolution generative adversarial network for PCD. The reported accuracy was 71% using 97.3% of available data as training data.

A combinatorial model was proposed using multiparametric magnetic resonance and a prostate health index with an artificial neural network algorithm for the recognition of prostate cancer [25]. The model achieved specificity of 68% and sensitivity of 80%. Recent research has detailed the roles of radiomics and genomics in disease management and risk stratification for prostate cancer management [26]. Radiomics increases the clinical value of prostate cancer management by converging the imaging derivate quantitative features, whereas genomics data are decoded and explained by radiomics.

### 1.2. Research Limitations of Existing Works

We observed several research limitations with existing works [17,18,19,20,21,22,23,24] that drove our research initiative for a new methodology for PCD.

- The whole benchmark datasets were not fully utilized in the model training and testing in some existing works [17,18,19,23,24];
- A single split validation (with either training and testing datasets, or training, testing, and validation datasets) was adopted in some existing works [17,19,21,22,23,24];
- The sensitivity and accuracy of the existing works [17,18,19,20,21,22,23,24] were less than 90%, which implied room for improvement of the PCD models;
- Biased classification was observed in [22,23] based on significant deviations between the sensitivity and specificity of the PCD models.

### 1.3. Research Contributions of Our Work

To address the abovementioned limitations, our work proposes a transfer learning-based multi-scale denoising convolutional neural network (TL-MSDCNN) model. The general ideas are to utilize the whole benchmark datasets for performance evaluation and analysis of the PCD models, adopting 5-fold cross-validation, enhancing the sensitivity, specificity, and accuracy of the PCD models, and reducing the extent of biased classification of the PCD models. The concise research contributions are summarized as follows:

- TL not only borrows the domain knowledge from heterogeneous datasets (of the same domain, prostate cancer dataset) for the target model but also enhances the image noise suppression in the target model;
- MSDCNN takes the roles in image noise suppression, feature extraction, and PCD. It also is fine-tuned using TL;
- Compared with the existing works, the proposed TL-MSDCNN improves the sensitivity, specificity, and accuracy by more than 10% using various benchmark datasets;
- Ablation studies also showed average improvements of 2.80%, 3.30%, and 3.13%, in accuracy by using denoising, multi-scale scheme, and transfer learning, respectively.

To ensure a more comprehensive analysis, our work considers the whole benchmark datasets in performance evaluation and analysis and provides discussion on the results of PCD models using 5-fold cross-validation.

## 2. Benchmark Datasets and Methodology

The details of the four benchmark datasets are firstly summarized. This is followed by the methodology of the TL-MSDCNN, which comprises three modules related to the Gaussian noise insertion, the MSDCNN, and the TL algorithms.

### 2.1. Summary of the Benchmark Datasets

Four benchmark datasets, NaF Prostate [12], TCGA-PRAD [13], Prostate-3T [14], and PROSTATE-DIAGNOSIS [15], were retrieved for the performance evaluation and analysis of the proposed TL-MSDCNN algorithm. The details of the datasets including data type, size of the dataset, the number of participants, the number of studies, the number of series, and the number of images, are summarized in Table 1. Different data types may be utilized for PCD where the proposed TL-MSDCNN is a generic approach to intake various data types. In terms of the number of images (or size of the dataset), we can categorize the datasets into small-scale (Prostate-3T), medium-scale (TCGA-PRAD and PROSTATE-DIAGNOSIS), and large-scale (NaF Prostate). With the aid of transfer learning, domain knowledge can be transferred from different datasets (reducing the impact on the performance of the model with the size of the dataset).

### 2.2. Methodology of the Transfer Learning-Based Multi-Scale Denoising Convolutional Neural Network (TL-MSDCNN)

Image noise insertion is first applied to the images of the benchmark datasets before the training of the PCD models. This is followed by the design of the DCNN. TL is applied to fine-tune the trained DCNN model in a three-round manner.

#### 2.2.1. Gaussian Noise Insertion into Images

Adding noise in the images of the benchmark datasets utilizes advantages in (i) performance evaluation and analysis of the MSDCNN model, which is capable of image noise suppression; and (ii) facilitates learning more domain knowledge from the noisy images across different datasets so that the proposed TL-MSDCNN serves as a dual noise suppression algorithm.

Gaussian noise is introduced to all images of the benchmark datasets. In general, it is generated along with the electronic components; that is the reason why Gaussian noise is also named as electronic noise. The noise significantly affects the greyscale value of the images and thus may decrease the accuracy of the PCD model. The probability density function (PDF) is given by:

(1) $$p\left(I\right)=\frac{e^{\frac{-{\left(I-\bar{I}\right)}^{2}}{2\sigma^{2}}}}{\sigma \sqrt{2\pi }}$$

where I is the intensity, $\bar{I}$ is the mean, and σ is the standard deviation of I.

Inspired by [27,28,29], we ranged the settings of the percentages of the Gaussian noise as the noise insertion into images from 5 to 50%, with a step size of 5%. The percentage specifies the ratio of the standard deviation of the Gaussian noise versus the signal of the entire image.

#### 2.2.2. Multi-Scale Denoising Convolutional Neural Network (MSDCNN)

**Figure 1:**
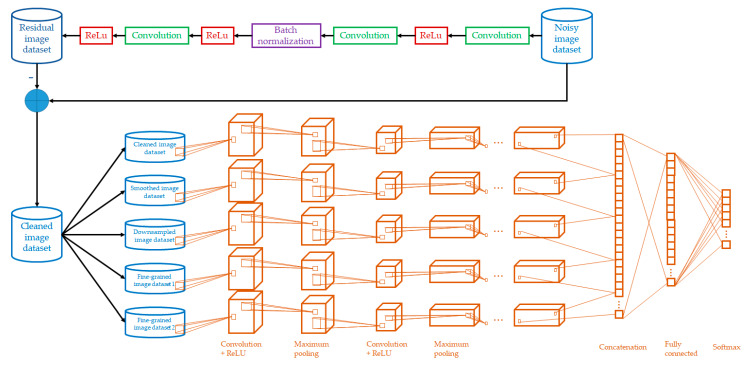
Architecture of the MSDCNN.

The architecture of the MSDCNN is shown in Figure 1. The algorithm can be divided into two parts: residual learning for image denoising and multi-scale convolutional neural network for the model training of the PCD. Each of the benchmark datasets follows the process of MSDCNN, which performs further transfer learning in next phase (Section 2.2.3).

The residual learning involves the process between the noisy image dataset and residual image dataset. To reduce the time complexity, it is formulated as a three-stage operation using two (Convolution and ReLu) operations and a (Convolution, batch normalization, and ReLu) operation. It was evaluated and confirmed in some works [30,31]. Another well-known image denoising approach is autoencoder. Recently, denoising autoencoder [32] and convolutional denoising autoencoder [33] were proposed for image denoising. The rationale of these algorithms was to learn denoised images from noisy images using several stacked layers. However, this type of approach experiences the issue of inability to effectively manage unseen noise types (beyond model training) [34]. Therefore, our work employs residual learning. Consider the fundamental formulation:

(2) $$I_{noisy}=I_{original}+z$$

where $I_{noisy}$ is the noisy image, $I_{original}$ is the original image, and *z* is some noise. The goal of the residual learning is to learn the image residue $I_{residue}$ to find the approximately cleaned image $I_{cleaned}$.

(3) $$I_{cleaned}=I_{noisy}-I_{residue}$$

For the batch normalization, assume that a batch of *N* input images $I=\left\{I_{1},\ldots ,I_{N}\right\}$ is introduced to the first layer of the model with variance $\sigma_{k}^{2}$. The dimension of the images will be normalized by:

(4) $$\hat{I_{k}}=\left(I_{k}-E\left(I_{k}\right)\right)/\sqrt{\sigma_{k}^{2}}$$

The output of the residual learning forms the cleaned image dataset, which is further processed using a multi-scale convolutional neural network. In the literature, there are two common designs for (i) the multi-scale smoothing and downsampling of images to form a smoothed image dataset and downsampled image dataset, respectively [35]; and (ii) fine-graining of the images to two more versions to create different granularities to form fine-grained image dataset 1 and fine-grained image dataset 2 [36]. In order to enhance the benefits of the multi-scale convolutional neural network, we propose to transform the cleaned image dataset with smoothing, downsampling, and fine-graining. In total, five datasets are used in the convolutional neural network in parallel with major components: convolution layers, ReLUs, and maximum pooling layers. The results for each dataset are first concatenated. This is followed by a fully connected layer and a softmax function.

**Figure 2:**
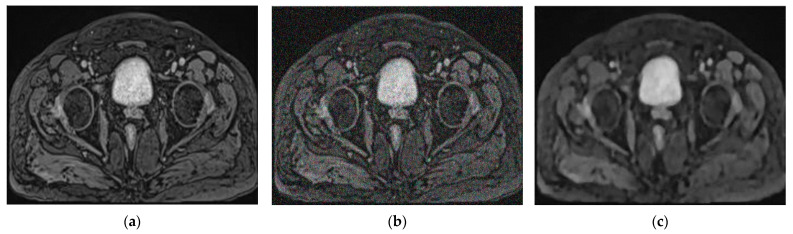
Examples of MRI images (**a**) original; (**b**) with Gaussian noise; (**c**) after applying residual learning.

Figure 2 shows some examples of MRI images in three versions: original, with Gaussian noise, and after applying residual learning.

#### 2.2.3. Transfer Learning (TL)

We considered the one-to-one transfer learning, which is the most robust approach to control the hyperparameters for the knowledge transfer from a pre-trained model to a target model. Recall that four benchmark datasets were selected for the performance evaluation and analysis of the TL-MSDCNN algorithm, and 12 target models were built, the details of which are summarized in Table 2. For easier understanding, we denote the model with subscripts for TL-MSDCNN using the in-text citations for the source and target datasets.

**Figure 3:**
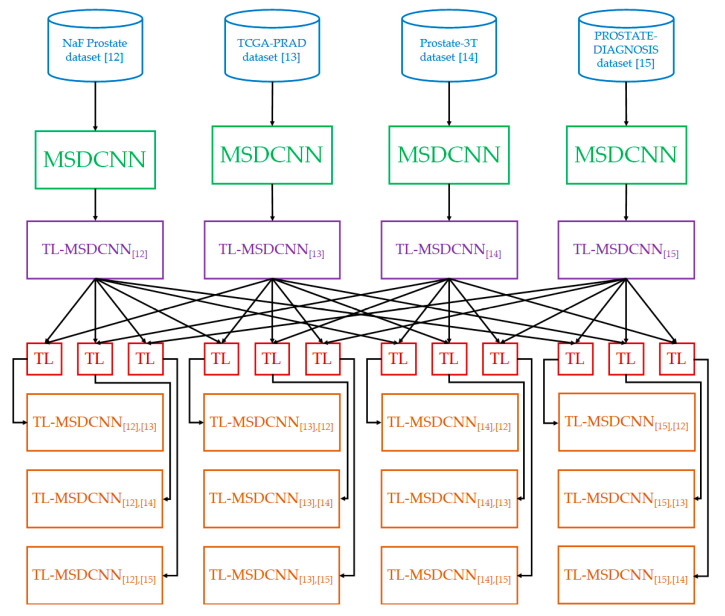
Architecture of the transfer learning with MCDCNN.

Figure 3 shows the architecture of the transfer learning with MCDCNN. The pre-trained models for the four benchmark datasets (TL-MSDCNN [12], TL-MSDCNN [13], TL-MSDCNN [14], and TL-MSDCNN [15]) with MSDCNN were obtained. One of the pre-trained models served as the source model to fine-tune the target model. As a result, 12 target models were built.

## 3. Performance Evaluation and Comparisons

To evaluate the performance of the TL-MSDCNN, a k-fold cross-validation was adopted that takes advantage of better examination of the issue of over-fitting, thus reducing its impact. Based on existing works [37,38,39], k = 5 was chosen. The performance evaluation metrics were the average of the sensitivity, specificity, and accuracy. The formulas are defined as follows:

(5) $$Sensitivity=(\overset{5}{\underset{i=1}{\sum }}\frac{TP_{i}}{TP_{i}+FN_{i}})/5$$

(6) $$Specificity=(\overset{5}{\underset{i=1}{\sum }}\frac{TN_{i}}{TN_{i}+FP_{i}})/5$$

(7) $$Accuracy=\omega_{1}Sensitivity+\omega_{2}Specificity$$

where $TP_{i}$, $TN_{i}$, $FP_{i}$, and $FN_{i}$ are the true positive rate, true negative rate, false positive rate, and false negative rate in the *i*-th fold, respectively. The weighting factors for the sensitivity and specificity are $\omega_{1}$ and $\omega_{2}$, respectively.

### 3.1. Performance Evaluation of TL-MSDCNN

Table 3 summarizes the average sensitivity, specificity, and accuracy of the 12 target models using TL-MSDCNN with and without Gaussian noise insertion. The model experienced more challenge when extra Gaussian noise was inserted in the prostate cancer images. Various observations are highlighted as follows.

- Taking averages of the metrics of three versions of each target model with Gaussian noise insertion, the average sensitivity, specificity, and accuracy were 95.8, 96.6, and 96.1% for NaF Prostate [12], 94.6, 95.4, and 94.9% for TCGA-PRAD [13], 98.3, 99.1, and 98.7% for Prostate-3T [14], and 95.5, 94.8, and 95.1% for PROSTATE-DIAGNOSIS [15];
- Likewise, without Gaussian noise insertion, the average sensitivity, specificity, and accuracy were 96.1, 96.9, and 96.4% for NaF Prostate [12], 94.8, 95.8, and 95.3% for TCGA-PRAD [13], 98.5, 99.3, and 98.9% for Prostate-3T [14], and 95.9, 95.2, and 95.5% for PROSTATE-DIAGNOSIS [15];
- The best target models with Gaussian noise insertion of each benchmark dataset were TL-MSDCNN _[15],[12]_ with result metrics of 96.8, 97.7, and 97.1% for NaF Prostate [12], TL-MSDCNN _[15],[13]_ with result metrics of 95.4, 96.3, and 95.8% for TCGA-PRAD [13], TL-MSDCNN _[15],[14]_ with result metrics of 98.9, 99.6, and 99.2% for Prostate-3T [14], and TL-MSDCNN _[13],[15]_ with result metrics of 96.9, 96.2, 96.6% for PROSTATE-DIAGNOSIS [15];
- Likewise, the best target models without Gaussian noise insertion of each benchmark dataset were TL-MSDCNN _[15],[12]_ with result metrics of 97.1, 98.0, and 97.4% for NaF Prostate [12], TL-MSDCNN _[15],[13]_ with result metrics of 95.8, 96.7, and 96.2% for TCGA-PRAD [13], TL-MSDCNN _[15],[14]_ with result metrics of 99.1, 99.7, and 99.3% for Prostate-3T [14], and TL-MSDCNN _[13],[15]_ with result metrics of 97.3, 96.5, and 96.9% for PROSTATE-DIAGNOSIS [15].

**Table 3:** Performance of the 12 target models using TL-MSDCNN with and without Gaussian noise insertion.

	With/Without Gaussian Noise Insertion		
Model	Average Sensitivity (%)	Average Specificity (%)	Average Accuracy (%)
TL-MSDCNN _[12],[13]_	94.6/94.9	95.3/95.7	94.9/95.2
TL-MSDCNN _[12],[14]_	97.5/97.7	98.4/98.7	98.1/98.3
TL-MSDCNN _[12],[15]_	95.3/95.6	94.7/95.0	94.9/95.2
TL-MSDCNN _[13],[12]_	95.7/95.9	96.5/96.8	96.0/96.3
TL-MSDCNN _[13],[14]_	98.6/98.8	99.2/99.4	98.9/99.1
TL-MSDCNN _[13],[15]_	96.9/97.3	96.2/96.5	96.6/96.9
TL-MSDCNN _[14],[12]_	94.9/95.3	95.6/95.9	95.2/95.5
TL-MSDCNN _[14],[13]_	93.8/94.2	94.5/94.9	94.1/94.5
TL-MSDCNN _[14],[15]_	94.2/94.7	93.6/94.0	93.9/94.3
TL-MSDCNN _[15],[12]_	96.8/97.1	97.7/98.0	97.1/97.4
TL-MSDCNN _[15],[13]_	95.4/95.8	96.3/96.7	95.8/96.2
TL-MSDCNN _[15],[14]_	98.9/99.1	99.6/99.7	99.2/99.3

### 3.2. Performance Comparison between TL-MSDCNN and Existing Works

The proposed TL-MSDCNN algorithm was compared with the existing works. It is noted that only the best TL-MSDCNN model of each dataset was chosen for the comparison. Table 4 compares the works in terms of cross-validation type, average sensitivity, specificity, and accuracy.

The following observations were drawn.

- The works either adopted 5-fold cross-validation or no cross-validation (simple training and testing datasets);
- Although the performance evaluation metrics (average sensitivity, specificity, or average accuracy) were not ready in some works, comparisons could be made with other non-zero metrics. Particularly, biased classification towards the cancer type or healthy type did not exist because of the sufficient data in all classes;
- The proposed TL-MSDCNN algorithm achieved the best results in all benchmark datasets. The ranges of improvement in terms of average sensitivity, specificity, and accuracy, respectively, were 10, 9.78, and N/A% for NaF Prostate [12], 17.1, 17.4, and 17.1–24.4% for TCGA-PRAD [13], 11.5–11.9, 0.505–6.64, and 0.507–7.83% for Prostate-3T [14], and N/A, N/A, and 22.3–36.1% for PROSTATE-DIAGNOSIS [15].

## 4. Ablation Studies

To reveal the effectiveness of the components of the TL-MSDCNN algorithm, ablation studies were conducted based on the removal of the image denoising algorithm, multi-scale scheme, and transfer learning. Ablation studies are useful to investigate the performance of an artificial intelligence system by eliminating a component to study its benefit to the whole system.

### 4.1. Image Denoising Algorithm

Table 5 compares the performance of the 12 target models with and without the image denoising algorithm (upper part of Figure 1). Taking the average of the metrics for three versions of each target model, the improvements of the proposed algorithm in terms of average sensitivity, specificity, and accuracy, respectively, were 2.83, 2.69, and 2.79% for NaF Prostate [12], 2.53, 2.69, and 2.63% for TCGA-PRAD [13], 2.22, 2.24, and 2.21% for Prostate-3T [14], and 3.57, 3.54, and 3.55% for PROSTATE-DIAGNOSIS [15].

### 4.2. Multi-Scale Scheme

Table 6 compares the performance of the 12 target models with and without multi-scale scheme, i.e., removing the four datasets, namely the smoothed image dataset, downsampled image dataset, fine-grained dataset 1, and fine-grained dataset 2 in the architecture of Figure 1. Taking the average of the metrics for three versions of each target model, the improvements of the proposed algorithm in terms of average sensitivity, specificity, and accuracy, respectively, were 3.75, 3.20, and 3.52% for NaF Prostate [12], 3.58, 3.29, and 3.44% for TCGA-PRAD [13], 3.33, 3.05, and 3.22% for Prostate-3T [14], and 2.80, 3.12, and 3.03% for PROSTATE-DIAGNOSIS [15].

To further analyze the ability of the TL-MSDCNN algorithm with noisy images, Gaussian smoothing with varying degrees of smoothing (standard deviation from 0.5 to 2.0 with step size of 0.25) was analyzed. Table 7 compares the performance of the 12 target models with image denoising algorithm between Gaussian noise and Gaussian smoothing approaches. Taking the average of the metrics for three versions of each target model, the models were more efficient with Gaussian noise compared with Gaussian smoothing. The improvements with Gaussian noise in terms of the average sensitivity, specificity, and accuracy, respectively, were 0.703, 0.838, and 0.736% for NaF Prostate [12], 0.710, 0.740, and 0.724% for TCGA-PRAD [13], 0.716, 0.711, and 0.713% for Prostate-3T [14], and 0.702, 0.671, and 0.686% for PROSTATE-DIAGNOSIS [15].

**Table 6:** Performance of the 12 target models using TL-MSDCNN when Gaussian noise and Gaussian smoothing are considered.

	With Gaussian Noise/With Gaussian Smoothing		
Model	Average Sensitivity (%)	Average Specificity (%)	Average Accuracy (%)
TL-MSDCNN _[12],[13]_	94.6/94.1	95.3/94.6	94.9/94.3
TL-MSDCNN _[12],[14]_	97.5/97.1	98.4/97.9	98.1/97.5
TL-MSDCNN _[12],[15]_	95.3/94.6	94.7/93.9	94.9/94.1
TL-MSDCNN _[13],[12]_	95.7/95.1	96.5/95.8	96.0/95.4
TL-MSDCNN _[13],[14]_	98.6/97.7	99.2/98.4	98.9/98.0
TL-MSDCNN _[13],[15]_	96.9/96.1	96.2/95.5	96.6/95.8
TL-MSDCNN _[14],[12]_	94.9/94.0	95.6/94.5	95.2/94.2
TL-MSDCNN _[14],[13]_	93.8/93.0	94.5/93.7	94.1/93.3
TL-MSDCNN _[14],[15]_	94.2/93.7	93.6/93.2	93.9/93.5
TL-MSDCNN _[15],[12]_	96.8/96.3	97.7/97.1	97.1/96.6
TL-MSDCNN _[15],[13]_	95.4/94.7	96.3/95.7	95.8/95.1
TL-MSDCNN _[15],[14]_	98.9/98.1	99.6/98.8	99.2/98.4

### 4.3. Transfer Learning

Table 8 compares the performance of the 12 target models with and without transfer learning. Taking the average of the metrics for three versions of each target model, the improvements of the proposed algorithm in terms of average sensitivity, specificity, and accuracy, respectively, were 3.12, 3.24, and 3.16% for NaF Prostate [12], 3.16, 3.32, and 3.23% for TCGA-PRAD [13], 2.86, 2.77, and 2.81% for Prostate-3T [14], and 3.28, 3.41, and 3.33% for PROSTATE-DIAGNOSIS [15].

## 5. Conclusions and Future Research Directions

To enhance the performance of the automatic diagnosis of prostate cancer, this paper proposes a transfer learning-based multi-scale denoising convolutional neural network (TL-MSDCNN) model. In several comparisons with existing works, our model improved the accuracy by more than 10%. Ablation studies also showed average improvements in accuracy using denoising, multi-scale scheme, and transfer learning by 2.80%, 3.30%, and 3.13%, respectively. It is understood that there is room for improvement in our research work. We suggest future research directions with the ideas of (i) investigating the effectiveness of the heterogeneous datasets of different disciplines to enhance the knowledge transfer between source and target models [40,41]; (ii) investigating the extent of smoothing, downsampling, and fine-graining of the multi-scale scheme on the performance of the model; (iii) generating additional training data using the variants of generative adversarial networks [42,43] because downsampling sacrifices the available ground truth data [44]; (iv) generating other types of noise such as speckle noise and random noise in the images to study the robustness of the model [45,46]; and (v) evaluating more noise injection approaches such as rotation, cropping, and re-sizing.

## Figures and Tables

**Table 1:** Summary of the benchmark datasets.

	Dataset			
Details	NaF Prostate [12]	TCGA-PRAD [13]	Prostate-3T [14]	PROSTATE-DIAGNOSIS [15]
Data type	PET/CT	MR, PT, CT	MR (T2W)	MR (T1, T2, and DCE sequences)
Size of the dataset (GB)	12.9	3.74	0.277	5.6
The number of participants	9	14	64	92
The number of studies	44	20	64	92
The number of series	214	207	64	368
The number of images	64,535	16,790	1258	32,537

**Table 2:** Details of the target models.

Model	Source Model	Target Model
TL-MSDCNN _[12],[13]_	NaF Prostate [12]	TCGA-PRAD [13]
TL-MSDCNN _[12],[14]_	NaF Prostate [12]	Prostate-3T [14]
TL-MSDCNN _[12],[15]_	NaF Prostate [12]	PROSTATE-DIAGNOSIS [15]
TL-MSDCNN _[13],[12]_	TCGA-PRAD [13]	NaF Prostate [12]
TL-MSDCNN _[13],[14]_	TCGA-PRAD [13]	Prostate-3T [14]
TL-MSDCNN _[13],[15]_	TCGA-PRAD [13]	PROSTATE-DIAGNOSIS [15]
TL-MSDCNN _[14],[12]_	Prostate-3T [14]	NaF Prostate [12]
TL-MSDCNN _[14],[13]_	Prostate-3T [14]	TCGA-PRAD [13]
TL-MSDCNN _[14],[15]_	Prostate-3T [14]	PROSTATE-DIAGNOSIS [15]
TL-MSDCNN _[15],[12]_	PROSTATE-DIAGNOSIS [15]	NaF Prostate [12]
TL-MSDCNN _[15],[13]_	PROSTATE-DIAGNOSIS [15]	TCGA-PRAD [13]
TL-MSDCNN _[15],[14]_	PROSTATE-DIAGNOSIS [15]	Prostate-3T [14]

**Table 4:** Performance comparison between TL-MSDCNN and existing works.

Dataset	Work	Type of Cross-Validation	Average Sensitivity (%)	Average Specificity (%)	Average Accuracy (%)
NaF Prostate [12]	[17]	No	88	89	N/A
	[18]	5-fold	88	N/A	N/A
	TL-MSDCNN _[15],[12]_	5-fold	96.8	97.7	97.1
TCGA-PRAD [13]	[19]	No	N/A	N/A	77
	[20]	5-fold	81.5	82	81.8
	TL-MSDCNN _[15],[13]_	5-fold	95.4	96.3	95.8
Prostate-3T [14]	[21]	No	88.4	93.4	92.0
	[22]	No	88.7	99.1	98.7
	TL-MSDCNN _[15],[14]_	5-fold	98.9	99.6	99.2
PROSTATE-DIAGNOSIS [15]	[23]	No	N/A	N/A	79
	[24]	No	N/A	N/A	71
	TL-MSDCNN _[13],[15]_	5-fold	96.9	96.2	96.6

**Table 5:** Performance of the 12 target models using TL-MSDCNN with and without image denoising algorithm when Gaussian noise is considered.

	With/Without Image Denoising Algorithm		
Model	Average Sensitivity (%)	Average Specificity (%)	Average Accuracy (%)
TL-MSDCNN _[12],[13]_	94.6/92.3	95.3/92.8	94.9/92.5
TL-MSDCNN _[12],[14]_	97.5/95.6	98.4/96.4	98.1/96.1
TL-MSDCNN _[12],[15]_	95.3/92.1	94.7/91.4	94.9/91.7
TL-MSDCNN _[13],[12]_	95.7/93.1	96.5/94.1	96.0/93.4
TL-MSDCNN _[13],[14]_	98.6/96.8	99.2/97.5	98.9/97.2
TL-MSDCNN _[13],[15]_	96.9/94.0	96.2/93.4	96.6/93.8
TL-MSDCNN _[14],[12]_	94.9/92.1	95.6/92.9	95.2/92.4
TL-MSDCNN _[14],[13]_	93.8/91.5	94.5/92.0	94.1/91.7
TL-MSDCNN _[14],[15]_	94.2/90.5	93.6/90.0	93.9/90.3
TL-MSDCNN _[15],[12]_	96.8/94.3	97.7/95.2	97.1/94.6
TL-MSDCNN _[15],[13]_	95.4/93.0	96.3/93.8	95.8/93.3
TL-MSDCNN _[15],[14]_	98.9/96.2	99.6/96.8	99.2/96.5

**Table 7:** Performance of the 12 target models using TL-MSDCNN with and without multi-scale scheme.

	With/Without Multi-Scale Scheme		
Model	Average Sensitivity (%)	Average Specificity (%)	Average Accuracy (%)
TL-MSDCNN _[12],[13]_	94.6/91.3	95.3/92.5	94.9/91.8
TL-MSDCNN _[12],[14]_	97.5/94.7	98.4/95.6	98.1/95.2
TL-MSDCNN _[12],[15]_	95.3/93.6	94.7/92.4	94.9/92.8
TL-MSDCNN _[13],[12]_	95.7/92.4	96.5/93.3	96.0/92.7
TL-MSDCNN _[13],[14]_	98.6/95.5	99.2/96.0	98.9/95.8
TL-MSDCNN _[13],[15]_	96.9/93.8	96.2/93.2	96.6/93.5
TL-MSDCNN _[14],[12]_	94.9/91.5	95.6/93.2	95.2/92.3
TL-MSDCNN _[14],[13]_	93.8/90.2	94.5/91.0	94.1/90.6
TL-MSDCNN _[14],[15]_	94.2/91.2	93.6/90.3	93.9/90.7
TL-MSDCNN _[15],[12]_	96.8/93.1	97.7/94.3	97.1/93.5
TL-MSDCNN _[15],[13]_	95.4/92.5	96.3/93.5	95.8/92.9
TL-MSDCNN _[15],[14]_	98.9/95.3	99.6/96.8	99.2/95.9

**Table 8:** Performance of the 12 target models using TL-MSDCNN with and without transfer learning.

	With/Without Transfer Learning		
Model	Average Sensitivity (%)	Average Specificity (%)	Average Accuracy (%)
TL-MSDCNN _[12],[13]_	94.6/91.8	95.3/92.2	94.9/92
TL-MSDCNN _[12],[14]_	97.5/94.6	98.4/95.6	98.1/95.2
TL-MSDCNN _[12],[15]_	95.3/92.1	94.7/91.4	94.9/91.7
TL-MSDCNN _[13],[12]_	95.7/92.8	96.5/93.6	96.0/93.2
TL-MSDCNN _[13],[14]_	98.6/95.9	99.2/96.5	98.9/96.2
TL-MSDCNN _[13],[15]_	96.9/93.6	96.2/92.8	96.6/93.1
TL-MSDCNN _[14],[12]_	94.9/92.3	95.6/92.9	95.2/92.5
TL-MSDCNN _[14],[13]_	93.8/90.7	94.5/91.4	94.1/90.9
TL-MSDCNN _[14],[15]_	94.2/91.6	93.6/90.9	93.9/91.2
TL-MSDCNN _[15],[12]_	96.8/93.6	97.7/94.2	97.1/93.8
TL-MSDCNN _[15],[13]_	95.4/92.6	96.3/93.3	95.8/92.9
TL-MSDCNN _[15],[14]_	98.9/96.3	99.6/97.1	99.2/96.7

## Data Availability

Not applicable.
